# Vitamin D as a Modulator of Sarcopenia in Women: Interactions with Dietary Patterns and Physical Exercise

**DOI:** 10.3390/nu18152494

**Published:** 2026-08-02

**Authors:** Ilaria Versari, Alberto Bavelloni, Camilla Sbrighi, Mirko Traversari, Irene Faenza, Sara Salucci

**Affiliations:** 1 Department of Biomedical and NeuroMotor Sciences (DIBINEM), University of Bologna, 40126 Bologna, Italy; ilaria.versari4@unibo.it (I.V.); camilla.sbrighi@irst.emr.it (C.S.); 2 Laboratory of Experimental Oncology, IRCCS, Istituto Ortopedico Rizzoli, 40136 Bologna, Italy; alberto.bavelloni@ior.it; 3 Biosciences Laboratory, IRCCS Istituto Romagnolo Per Lo Studio Dei Tumori (IRST) “Dino Amadori”, 47014 Meldola, Italy; 4 Department of Medical and Surgical Sciences (DIMEC), University of Bologna, 40126 Bologna, Italy; mirko.traversari2@unibo.it

**Keywords:** vitamin D, skeletal muscle tissue, postmenopausal women, nutritional interventions, physical exercise

## Abstract

Women are particularly vulnerable to sarcopenia- a progressive, age-related condition characterized by the loss of skeletal muscle mass, strength, and physical performance- due to sex-specific hormonal and physiological changes, most notably the decline in estrogen levels after menopause. This increased risk highlights the critical importance of developing tailored, sex-specific strategies for prevention, diagnosis, and management. Among nutritional factors, vitamin D is increasingly recognized as a key regulator of skeletal muscle physiology, exerting direct effects on muscle tissue via vitamin D receptors and modulating the gene expression involved in muscle cell proliferation, protein synthesis, and mitochondrial function. This review aims to evaluate the synergistic roles of vitamin D, nutrition, and exercise in improving skeletal muscle function in women, with a specific focus on the prevention and management of sarcopenia. Vitamin D can contribute to skeletal muscle integrity by influencing fiber composition, preserving type II fibers, and preventing mass loss. Additionally, it promotes satellite cell activation, thereby supporting muscle repair and regeneration. Consequently, vitamin D supplementation can improve muscle strength, balance, and physical performance, while reducing the risk of falls, frailty, osteoporosis and sarcopenia. Recent studies suggest that vitamin D acts synergistically with diets enriched with whey protein and leucine, as well as with structured exercise programs, predominantly resistance training, to further enhance muscle function and bone health. This review critically examines the current evidence regarding these combined interventions in preserving and optimizing skeletal muscle function in older women, while also addressing the major limitations and inconsistencies in available studies. Understanding the collective impact of these strategies is crucial for developing personalized and effective strategies aimed at maintaining musculoskeletal health, physical performance, and functional independence throughout the female aging process.

## 1. Introduction

### 1.1. Sarcopenia in Women

Sarcopenia is a progressive and generalized skeletal muscle disorder characterized by a loss of muscle mass, strength, and physical performance. It is increasingly recognized as a major public health concern in aging populations [1,2]. Sarcopenia, defined as the age-related loss of muscle mass and function [3], is characterized by distinct structural alterations. These include a reduction in both the size and quantity of myofibers, as well as impairments in satellite cell density and functionality. Consequently, this condition is associated with adverse outcomes such as physical disability, a poor quality of life, and increased mortality. The clinical diagnosis of sarcopenia has evolved significantly over the last decade, transitioning from a definition purely based on muscle mass depletion to a more functional, multi-component approach. Several consensus groups have established specific diagnostic algorithms, with the European Working Group on Sarcopenia in Older People (EWGSOP) serving as a primary reference [4].

Under the EWGSOP2 framework, muscle strength is now recognized as the principal determinant of sarcopenia. A diagnosis is considered probable when low muscle strength is detected (measured via handgrip strength or the chair stand test). The diagnosis is confirmed by the additional documentation of low muscle quantity or quality and is classified as severe if these criteria are accompanied by poor physical performance [4].

Although sarcopenia affects both sexes, accumulating evidence suggests that women may be disproportionately impacted due to sex-specific physiological and hormonal factors. The decline in estrogen levels during menopause accelerates muscle degradation [5,6]. This hormone reduction induces oxidative stress across various tissues, including skeletal muscle, thereby impairing muscle protein synthesis and triggering mitochondrial dysfunction [7]. The study by Blumel and colleagues highlighted a striking age-related decline in muscle strength: while only 7.1% of women in their 40s exhibited low muscle strength, this prevalence rose dramatically to 79.4% among women in their 80s, with more than half of older women meeting the clinical criteria for sarcopenia [8]. Crucially, sarcopenia is a multifactorial condition involving chronic inflammation, nutritional deficiencies, and reduced physical activity [9]. In women, these mechanisms are often compounded by a lower baseline muscle mass and a higher prevalence of phenotypes such as osteosarcopenic obesity, which further increase vulnerability to frailty and metabolic dysfunction [10].

Over the past five years, advances in research have improved diagnostic criteria, incorporating the development of more precise muscle mass cut-offs and functional assessments tailored to different populations. In addition, growing evidence supports the role of combined interventions, such as resistance training and protein supplementation, in sarcopenia prevention [11,12]. Despite this progress, sarcopenia in women remains underdiagnosed and undertreated, highlighting the need for increased awareness and targeted preventive strategies.

### 1.2. Nutritional Factors

In recent years, accumulating studies have underscored the multifactorial etiology of sarcopenia, positioning nutrition as a crucial modifiable determinant [3,13]. Inadequate dietary intake, particularly insufficient protein consumption, is consistently linked to the onset and progression of the condition. A recent systematic review and meta-analysis demonstrated that sarcopenic individuals exhibited significantly lower energy and nutrient intake compared to non-sarcopenic controls, highlighting the critical role of dietary adequacy in preserving muscle health [14,15].

Protein intake acts as a key factor in the regulation of muscle protein synthesis, particularly through essential amino acids such as leucine. However, aging is associated with anabolic resistance, requiring a higher protein intake to achieve the same muscle-building response [16]. Beyond protein, other nutrients such as vitamin D, omega-3 fatty acids, and antioxidants have been implicated in muscle metabolism, inflammation modulation, and mitochondrial function, all of which are relevant to the pathophysiology of sarcopenia [17,18,19].

In this review, vitamin D was selected from among various micronutrients due to its strong biological plausibility, its high prevalence of deficiency in women, and consistent association with sarcopenia-related outcomes, as highlighted in the recent literature. Unlike many other micronutrients, vitamin D exerts direct effects on skeletal muscle tissue through vitamin D receptors (VDRs), which are expressed in muscle cell myonuclei, where they regulate muscle protein synthesis, cell differentiation, and overall muscle function [20].

Furthermore, vitamin D deficiency is highly prevalent among women, particularly within older and postmenopausal cohorts. Age-related reductions in cutaneous synthesis, lower dietary intake, and hormonal shifts contribute to decreased circulating 25-hydroxyvitamin D levels. Given that women are already at a higher risk of sarcopenia due to lower baseline muscle mass and estrogen decline, this widespread hypovitaminosis represents a significant and modifiable risk factor [21,22].

Another compelling reason to focus on vitamin D is its pivotal role in neuromuscular function and inflammation, both of which drive the pathophysiology of sarcopenia. Beyond maintaining calcium homeostasis and muscle contraction efficiency, vitamin D can exert anti-inflammatory actions that mitigate the chronic, low-grade inflammation responsible for accelerated muscle degradation. Consequently, these multimodal effects place vitamin D at the nexus of the core pathophysiological mechanisms underlying female sarcopenia [23].

## 2. Materials and Methods

This paper is a narrative (non-systematic) literature review. Articles were selected based on their relevance to vitamin D, sarcopenia, nutrition, and exercise in women, without adhering to a formal systematic methodology or specific inclusion and exclusion criteria.

Specifically, this narrative review synthesizes current evidence on the role of vitamin D in managing sarcopenia among older and postmenopausal women. First, we provide an overview of sarcopenia in women and outline the rationale for focusing on vitamin D among the nutritional factors involved in muscle health. We then discuss vitamin D metabolism, circulating concentrations, and its specific relevance to female physiology. Particular attention is given to the role of vitamin D in skeletal muscle homeostasis and the molecular signaling pathways involved. Finally, we review the available evidence regarding the effects of vitamin D supplementation, both as a standalone intervention and when combined with other nutritional strategies and physical exercise programs.

To identify the relevant literature, a scoping search of PubMed, Scopus, and Web of Science was performed, focusing primarily on studies published between January 2011 and May 2026. The search was guided by keywords and Medical Subject Headings related to vitamin D, sarcopenia, skeletal muscle health, older and postmenopausal women, nutritional supplementation, and physical exercise. Relevant articles were selected based on their direct pertinence to the themes discussed in this review. Figures were created and prepared using BioRender (BioRender, Toronto, ON, Canada), ChatGPT-4o (OpenAI, San Francisco, CA, USA), and Adobe Photoshop CC 2018 (Adobe Inc., San Jose, CA, USA).

## 3. Vitamin D

**Figure 1:**
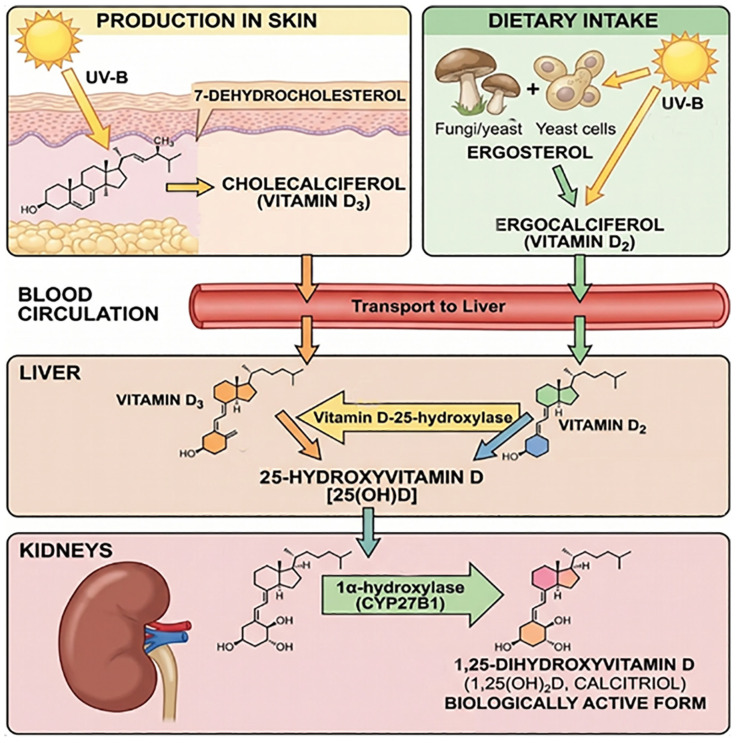
Synthesis of the active metabolite calcitriol from dietary intake and sunlight exposure. This figure was conceptualized using ChatGPT (ChatGPT-4o) and manually reconstructed and finalized using Adobe Photoshop.

Vitamin D is a fat-soluble vitamin, and its most biologically active form in humans is 1,25-dihydroxyvitamin D (1,25(OH)_2_D, calcitriol), which is primarily synthesized in the liver and kidneys and recognized as the main hormonal form of the vitamin [24]. The precursors to this biologically active form are ergocalciferol (vitamin D2) and cholecalciferol (vitamin D3). Vitamin D2 is synthesized in fungi and yeast through UV-B irradiation of the membrane sterols and is acquired by humans primarily through dietary intake. Conversely, vitamin D3 is produced endogenously in human skin when 7-dehydrocholesterol absorbs UV-B radiation, initiating its photochemical conversion to cholecalciferol [25]. Calcitriol is subsequently produced through two sequential hydroxylation reactions. In the liver, vitamin D-25-hydroxylase converts vitamin D into the main circulating form, 25-hydroxyvitamin D (25(OH)D), which is 1000-fold less potent than calcitriol [26]. The second hydroxylation is catalyzed by 1α-hydroxylase (CYP27B1), which generates the biologically active form [27]. Besides the kidneys, extrarenal CYP27B1 activity occurs in various tissues, such as skeletal muscle, the placenta, and epithelium. In these sites, resident immune cells, including macrophages and dendritic cells, express CYP27B1 to drive localized vitamin D activation during immune responses [28]. Figure 1 summarizes the key principles of vitamin D production and metabolism.

Vitamin D acts as a transcriptional regulator via its ability to bind to nuclear VDRs, which are predominantly expressed in skeletal muscle, bone, the kidneys, and the immune system. This widespread distribution underlies the pleiotropic physiological effects of vitamin D. Specifically, the vitamin D–VDR complex regulates the transcription of target genes involved in calcium and phosphate homeostasis, bone mineralization, and muscle cell proliferation and differentiation [27]. Vitamin D homeostasis is maintained by the enzyme 24-hydroxylase (CYP24A1), which controls the inactivation of both 25(OH)D and the active hormone calcitriol. Hormonal signals (including parathyroid hormone), cytokines, inflammatory stimuli, and pathological conditions can further modulate CYP24A1 activity, shifting the dynamic balance between the activation and degradation of vitamin D metabolites. To evaluate vitamin D status, serum levels of 25(OH)D are typically monitored. The threshold concentrations, recommended by the Institute of Medicine [29], are reported in Table 1.

This classification provides a useful framework for assessing vitamin D levels; however, these thresholds also reflect a continuum of biological and clinical risk, particularly in relation to skeletal muscle health and sarcopenia.

Optimal levels of vitamin D are associated with improved muscle protein synthesis, enhanced neuromuscular coordination, and a reduced risk of falls [30,31].

Adequate levels are generally considered sufficient for bone health and basic neuromuscular function. However, emerging evidence suggests that in older adults, especially postmenopausal women, this range may not fully optimize muscle strength or functional performance due to age-related anabolic resistance [32]. Insufficient levels typically represent a subclinical condition yet remain clinically relevant. Although individuals in this range do not present overt symptoms, evidence suggests they exhibit reduced muscle strength, slower gait speed, and impaired physical performance compared with those with an adequate status. This intermediate state may contribute to early sarcopenic changes through chronic low-grade inflammation and reduced muscle protein synthesis. Observational studies indicate a linear relationship between 25(OH)D levels and physical performance indicators, such as grip strength and gait speed [33,34]. Finally, deficient levels are consistently associated with clinically significant impairment in musculoskeletal function. At this stage, vitamin D deficiency disrupts calcium homeostasis and neuromuscular signaling, leading to muscle weakness, reduced physical performance, and an increased risk of falls [35].

### 3.1. Vitamin D Relevance in Women

In women, vitamin D status is a fundamental determinant of health outcomes across the lifespan, acting as a key pro-hormone with systemic influence. Beyond its traditional role in mineral metabolism, VDRs are widely expressed throughout the female reproductive system—including the ovaries, endometrium, and placenta—where they support critical processes such as folliculogenesis and oocyte quality, while potentially mitigating conditions like endometriosis [35,36,37,38,39,40,41,42]. The clinical significance of vitamin D becomes particularly evident during the postmenopausal transition. As estrogen levels decline, their protective effects on the skeleton diminish, leading to accelerated bone resorption. Emerging mechanistic evidence suggests a tight crosstalk between estrogen signaling and vitamin D action within tissues. Interestingly, studies on human and animal models have shown that 17-β-estradiol (E2) downregulates CYP24A1, promoting vitamin D accumulation and a consequently stronger anti-inflammatory response in females than in males [43]. Furthermore, E2 upregulates VDR gene expression across various human and rodent tissues, thus maintaining the adequate receptor density required for long-term vitamin D-mediated protein synthesis [43]. This close interplay was further evidenced by significantly elevated 25(OH)D levels in women using estrogen-containing contraceptives and the clinical association between low 25(OH)D and low E2 levels [43].

Therefore, maintaining optimal vitamin D concentration prevents severe deficiency states that would otherwise accelerate muscle wasting. Furthermore, vitamin D can act in synergy with nutritional or mechanical stimuli: while hormone depletion reduces baseline VDR expression, resistance exercise or protein intake robustly upregulates these receptors, restoring tissue sensitivity [11,12,42,43,44,45].

In this physiological context, vitamin D deficiency often triggers secondary hyperparathyroidism, which further alters bone metabolism, reduces bone mass, and increases the incidence of osteoporotic fractures [44]. Beyond bone density, muscle function serves as a critical indicator of physical health in women with osteopenia or osteoporosis. Preserving muscular strength is vital for preventing falls, reducing fracture risk, and maintaining overall quality of life in this at-risk population [44]. The essential role of vitamin D in musculoskeletal integrity was underscored by Feskanich and colleagues in 2003, who demonstrated that vitamin D supplementation significantly reduced osteoporotic hip fracture risk in postmenopausal women [45], a benefit subsequently confirmed by Liu and coworkers in 2020 [46]. Therefore, adequate vitamin D levels appear particularly important for preserving bone density, mitigating fracture risk, and supporting musculoskeletal function [47]. In this context, emerging evidence suggests that vitamin D can play a pivotal role in modulating skeletal muscle homeostasis. By targeting both the architectural (bone) and the mechanical (muscle) components of the musculoskeletal system, vitamin D may stand as a cornerstone of effective prevention and treatment strategies for sarcopenic women [48].

### 3.2. Sexual Dimorphism in Vitamin D Action and Sarcopenia

When examining the impact of vitamin D on musculoskeletal health, clinical and epidemiological studies frequently yield highly divergent results between men and women, demonstrating that its actions are heavily modulated by biological sex. Data indicate that low circulating serum 25(OH)D levels correlate with an accelerated decline in lower-extremity physical performance and muscle strength predominantly in older women, whereas these functional losses are often attenuated or non-significant in age-matched male cohorts [43]. This discrepancy is further reflected in the incidence of sarcopenia, where severe vitamin D insufficiency exhibits a robust, predictive association with accelerated myofibrillar mass depletion in postmenopausal women, contrasting with the far weaker or more inconsistent correlations observed in middle-aged and older men [49]. The underlying reasons for these sex-specific differences are rooted in distinct endocrine, metabolic, and anatomical profiles. Biologically, the abrupt postmenopausal withdrawal of estrogen E2 in women eliminates a primary stimulus for mitochondrial quality control and anti-inflammatory signaling, thereby upregulating catabolic cytokines, such as IL-6 and TNF-alpha. This heightened pro-inflammatory and catabolic state in women exacerbates skeletal muscle vulnerability, rendering myofibers significantly more sensitive to the structural damage caused by concurrent vitamin D deficiency. Conversely, older men maintain a more gradual, age-related decline in anabolic androgens (specifically testosterone and IGF-1), which continue to exert a protective, stabilizing effect on muscle protein synthesis, effectively buffering skeletal muscle against the negative impacts of low vitamin D [49]. Furthermore, these divergences are compounded by distinct body composition phenotypes; the inherently higher total body fat mass percentage in women facilitates the sequestration of fat-soluble vitamin D into adipose tissue, drastically reducing its active bioavailability for skeletal muscle cells across identical body mass index categories [43]. These discordant findings suggest that vitamin D action cannot be analyzed independently of sex steroid dynamics.

### 3.3. Vitamin D Signaling in Skeletal Muscle

Vitamin D is increasingly recognized as a key contributor to skeletal muscle biology [25]. Clinical and experimental evidence links its deficiency to muscle weakness, atrophy, impaired regeneration, and an increased risk of falls, particularly in the context of aging and disease [50,51]. These observations have driven growing interest in the molecular mechanisms through which vitamin D influences muscle structure and function. This role in skeletal muscle physiology is mediated by both genomic and non-genomic signaling pathways. Principally, vitamin D exerts its effects mainly via VDRs, expressed in skeletal muscle fibers and satellite cells, thus regulating protein synthesis [50], mitochondrial oxygen consumption [51], and myogenesis [52], as well as myocyte differentiation and proliferation [53]. In this tissue, the VDR acts as an essential molecular hub whose abundance depends on the differentiation state of the muscle cell. Indeed, VDR expression is higher in myoblasts than in myotubes, and increases markedly in regenerating muscle fibers and satellite cells following injury, indicating that vitamin D signaling is actively engaged during the repair process. Consistently, multiple studies in rodent models have shown that VDR expression is strongly upregulated in central myonuclei and activated satellite cells post-injury [54,55,56,57].

Vitamin D binds to the VDR, inducing a conformational change that allows for heterodimerization with the retinoid X receptor (RXR). Upon translocating to the nucleus, the VDR–RXR complex regulates the expression of genes crucial for myogenesis and muscle differentiation, including MyoD and myogenin [58]. Furthermore, it plays a key role in maintaining type II muscle fibers, calcium homeostasis, mitochondrial function, and oxidative metabolism [58]. Through these genomic effects, vitamin D supports muscle growth, structural integrity, and long-term functional performance [59]. Concurrently, the VDR can also trigger non-genomic signaling by modulating several intracellular pathways, such as MAPK, PI3K/Akt, and PKC, highlighting the rapid effects of this hormone on skeletal muscle homeostasis [30,60,61,62]. This direct interaction plays a critical role in regulating skeletal muscle protein synthesis and degradation. The main non-genomic signaling mechanisms triggered by vitamin D in skeletal muscle are outlined below.

#### 3.3.1. MAPK, PI3K/Akt, and PKC Pathways: Impact on Muscle Strength and Contractile Force

At the cellular level, the vitamin D–VDR complex modulates rapid non-genomic networks, including the Mitogen-Activated Protein Kinases (MAPK/ERK), Phosphoinositide 3-kinase (PI3K/Akt), and Protein Kinase C (PKC) pathways [63,64,65,66,67,68]. In primary human skeletal muscle cells, the activation of these cascades drives myoblast proliferation, stimulates the transition of satellite cells from a quiescent to an active regenerative state, and promotes downstream protein synthesis via mTORC1 activation [52,53]. Clinically, this molecular upregulation translates into preserved muscle quality and enhances contractile force. In postmenopausal women, the efficient activation of these pathways counteracts age-related anabolic resistance. Randomized controlled trials and systematic reviews confirm that the downstream physiological expression of these pathways manifests as significant improvements in handgrip strength, quadriceps power, and lower-limb functional extension. Furthermore, the rapid modulation of calcium influx supported by these pathways directly enhances neuromuscular coordination, providing a biological basis for the reduced risk of falls and fractures documented in older female cohorts [30,60].

#### 3.3.2. FOXO and IGF-1 Axes: Preservation of Muscle Mass and Counteracting Atrophy

Vitamin D signaling interacts closely with key anabolic and catabolic regulators, specifically upregulating the Insulin-like Growth Factor 1 (IGF-1) axis while simultaneously suppressing the Forkhead Box O (FOXO) family of transcription factors. Primary human cellular models indicate that VDR activation downregulates atrophy-related E3 ubiquitin ligases (such as MuRF1 and Atrogin-1), thereby minimizing proteolytic degradation and preserving myofibrillar structural integrity [66,69,70,71,72,73]. In clinical research, the balance between IGF-1 stimulation and FOXO inhibition directly correlates with the maintenance of lean mass and the prevention of myofibrillar wasting. Large-scale epidemiological data and randomization analyses indicate that severe vitamin D insufficiency predicts accelerated loss of lower-extremity lean mass predominantly in postmenopausal women. Clinical trials demonstrate that active vitamin D treatment or targeted cholecalciferol supplementation can mitigate appendicular skeletal muscle mass depletion, showing a potential protective effect against the onset of sarcopenic phenotypes in women [53,60].

#### 3.3.3. Inflammation, Oxidative Signaling, and Mitochondrial Homeostasis

Skeletal muscle aging in women is characterized by chronic low-grade inflammation and the accumulation of reactive oxygen species (ROS), which drive mitochondrial dysfunction and muscle wasting. The activation of the VDR serves as a key modulator of these processes by inhibiting the pro-inflammatory NF-κB pathway—consequently reducing catabolic cytokines like TNF-α and IL-6—and by promoting peroxisome proliferator-activated receptor gamma coactivator 1-alpha (PGC-1α) activity to support mitochondrial biogenesis and antioxidant defenses [28,74,75,76].

From a clinical perspective, managing this inflammatory and oxidative stress is vital for sustaining functional independence. Meta-analyses of randomized trials show that the suppression of systemic inflammatory markers via adequate vitamin D levels is linked to superior performance on objective physical tests, such as the Short Physical Performance Battery (SPPB) and timed stair-climbing assessments. Optimizing mitochondrial bioenergetics through these pathways helps preserve fast-twitch type II muscle fibers, directly improving gait speed, postural balance, and overall physical mobility in elderly women [53].

#### 3.3.4. Estrogen Signaling Crosstalk and Sexual Dimorphism

A unique aspect of female musculoskeletal pathophysiology is the intricate crosstalk between vitamin D and estrogen signaling. Estrogen receptors (ERs) localized in skeletal muscle optimize mitochondrial quality control and promote satellite cell self-renewal. Biochemical models suggest that the abrupt postmenopausal decline in E2 removes a primary stimulus for VDR stability, accelerating VDR desensitization and exacerbating catabolic pathways [43,44,45,46]. This molecular interplay explains the pronounced sexual dimorphism observed in clinical epidemiological studies. While older men are partially buffered against vitamin D deficiency by a more gradual decline in anabolic androgens, postmenopausal women exhibit a far tighter and more immediate correlation between low circulating 25(OH)D levels and accelerated functional decline. Although the exact molecular modifications under estrogen-deficient conditions await direct validation in human myotube models, clinical data strongly emphasize that maintaining optimal vitamin D status is a mandatory prerequisite to offset the musculoskeletal frailty accelerated by postmenopausal hormonal shifts [43].

**Figure 2:**
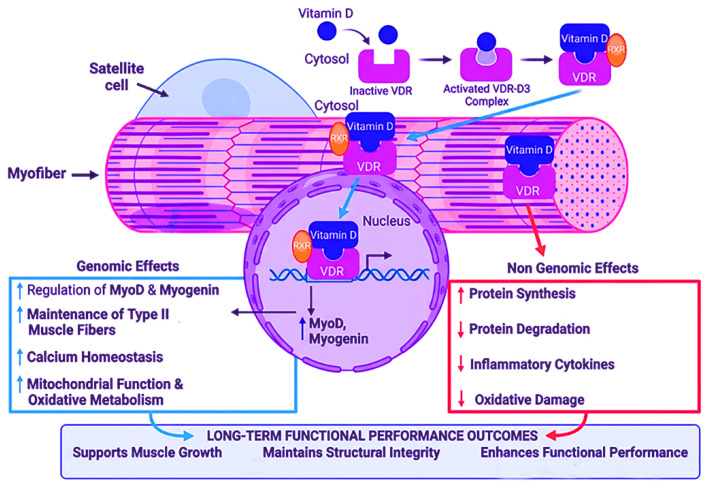
Genomic and non-genomic mechanisms of vitamin D action in skeletal muscle. This figure was created in BioRender. Salucci, S. (2026) https://app.biorender.com/illustrations/6a6c6d174ef729696e9d45beder.com and professionally refined using Adobe Photoshop.

Taken together, these genomic and non-genomic pathways establish vitamin D as a potential multifaceted regulator of skeletal muscle physiology, with significant implications for muscle health, regeneration, and homeostasis; the comprehensive mechanisms underlying these effects are schematically illustrated in Figure 2.

## 4. Vitamin D Supplementation in Sarcopenia

Among the multiple factors involved in sarcopenia pathogenesis, vitamin D deficiency has emerged as an important and potentially modifiable contributor. Reduced vitamin D levels are considered a common marker in female sarcopenic patients [50,77,78]. Epidemiological and interventional studies indicate that low serum 25(OH)D levels are associated with reduced muscle strength, slower gait speed, and a higher risk of sarcopenia in older populations, driven by diminished VDR expression and impaired activation of anabolic signaling pathways [79,80]. In addition, hypovitaminosis D is associated with increased myostatin expression, which negatively affects type II (fast-twitch) muscle fibers, which are essential for strength and balance. Furthermore, vitamin D deficiency impairs satellite cell activation, reduces mitochondrial biogenesis, and exacerbates inflammation and oxidative stress, thereby decreasing muscle quality and accelerating muscle wasting [50,54,55,56,57]. Overall, while vitamin D deficiency is not considered an independent cause of sarcopenia, it represents a significant contributing factor that accelerates muscle loss in older adults, especially within the framework of aging, sedentary lifestyle, and chronic disease. Sarcopenia is particularly prevalent among postmenopausal women, in whom the coexistence of hormonal alterations, especially estrogen decline and vitamin D deficiency, negatively impacts muscle metabolism, homeostasis, and functional integrity [21].

According to the International Osteoporosis Foundation, a dietary vitamin D intake of 20–25 µg/day (800–1000 IU/day) is recommended to reduce the risk of falls and fractures and may contribute to the prevention and management of sarcopenia in older women. In fact, observational evidence shows that postmenopausal women with lower levels of 25(OH)D tend to have reduced muscle strength, lower muscle mass, and poorer physical performance [57,81,82]. A randomized controlled trial in postmenopausal women indicated that vitamin D3 supplementation can increase muscle strength and help to maintain lean mass compared with placebo [83]. Vitamin D3 was administered to younger postmenopausal women aged 50 to 65 years who had experienced the absence of a menstrual period for at least 12 months. This population was divided into two groups: a supplemented group, consisting of patients receiving vitamin D3 supplementation (*n*  =  80), and a placebo group, consisting of patients receiving a placebo (*n*  =  80). Notably, a significant mitigation of lean mass loss alongside a 23.5% increase in lower limb muscle strength was observed in the vitamin D3 group, underlining its potential role as a protective factor against sarcopenia [83]. Beaudart and colleagues demonstrated that vitamin D supplementation significantly improved muscle strength, with a greater effect observed in individuals (including postmenopausal women and older postmenopausal women) with serum 25(OH)D concentrations below 75 nmol/L, compared to those with levels above 75 nmol/L [84]. This systematic review found that vitamin D supplementation improved handgrip strength, defined as the amount of force a muscle can produce, measured by grip strength, quadriceps muscle strength, and leg extension strength. This occurred particularly in women over 60 and in those with higher baseline vitamin D levels, although effects on muscle mass were not significant [84]. These positive effects on muscle strength may help to explain the beneficial impact of vitamin D on fall risk. However, vitamin D supplementation appeared to have no significant effect on muscle mass. This last finding should be interpreted with caution, given the limited number of studies available [84]. A meta-analysis of 13 trials confirmed a significant effect of vitamin D supplementation on muscle strength in postmenopausal women [32]. Furthermore, a randomized, double-blind, placebo-controlled trial by Kawahara and colleagues [85] evaluated the effect of eldecalcitol (0.75 μg/day), an active vitamin D analog, on sarcopenia prevention in adults with prediabetes who were free of sarcopenia at baseline. Sarcopenia was assessed using appendicular skeletal muscle mass and handgrip strength, and the incidence of sarcopenia was significantly lower in the eldecalcitol group than in the placebo group (4.6% vs. 8.8%). The protective effect was particularly evident in women, suggesting that active vitamin D may help preserve muscle mass and strength and reduce the risk of sarcopenia in this population [85]. Another randomized, placebo-controlled trial evaluated the effects of daily supplementation with 20 μg calcifediol or 3200 IU vitamin D3 on lower-extremity muscle function in 152 postmenopausal women aged 50–70 years with osteopenia or osteoporosis and baseline serum 25(OH)D concentrations below 75 nmol/L. Compared with placebo, both interventions improved vitamin D status, although calcifediol produced a more rapid increase in serum 25(OH)D levels [86]. While the study assessed lower-limb muscle performance through functional measures to evaluate how optimizing vitamin D status may contribute to preserving function in postmenopausal women at risk of musculoskeletal decline, these biochemical improvements did not translate into statistically significant functional enhancements. This lack of efficacy may be attributable, at least in part, to the unexpected rise in serum 25(OH)D levels within the placebo group, which potentially upregulated VDR expression and consequently attenuated the detectable effects of vitamin D supplementation on skeletal muscle [86]. Conversely, previous meta-analyses have yielded conflicting data, reporting insignificant or limited benefits of vitamin D supplementation on muscle mass and strength in postmenopausal women or the older female population, suggesting that vitamin D alone is often insufficient to delay sarcopenia [85,87,88,89]. Overall, evidence regarding the effects of vitamin D supplementation on muscle strength and physical function remains controversial, with multiple studies failing to demonstrate significant improvements in female sarcopenic cohorts. This may be due to confounding methodological factors, including the absence of severe baseline vitamin D deficiency, the heterogeneity of the study populations, relatively short intervention periods, and the lack of concomitant anabolic stimuli such as resistance exercise. Therefore, further investigations in female populations are warranted to clarify these discrepancies and to better define the role of vitamin D in maintaining skeletal muscle health in women.

## 5. Vitamin D Plus Nutritional Intervention Against Sarcopenia

Combined supplementation with vitamin D and muscle-supporting nutrients, particularly whey proteins, leucine, and essential micronutrients, appears to be especially crucial for older and postmenopausal women. This population is more vulnerable to sarcopenia due to hormonal decline, chronic inflammation, and reduced anabolic sensitivity [90,91]. Leucine and whey protein play a central role in skeletal muscle anabolism by activating key nutrient-sensing and protein synthesis pathways that converge on the mTORC1 signaling complex. Leucine acts as a direct molecular trigger of mTORC1 through intracellular amino acid–sensing mechanisms involving Sestrin2, GATTOR2/1 and Rag GTPases, leading to the activation of downstream effectors such as p70S6 kinase and 4E-BP1, which promote muscle protein synthesis. Whey protein, due to its rapid digestibility and high leucine content, induces a rapid surge in circulating amino acids and insulin secretion, thereby stimulating IGF-1 and the PI3K/Akt pathway. This dual activation enhances mTORC1 signaling while inhibiting the FOXO-mediated transcription of muscle atrophy genes, such as MuRF1 and Atrogin-1, ultimately reducing proteolysis and improving net protein balance [92].

Verlaan and coworkers [90] evaluated 380 community-dwelling sarcopenic older adults (men and women aged ≥65 years) who participated in a 13-week randomized, double-blind trial of a leucine-enriched whey protein supplement containing vitamin D (800 IU/day). The authors found that participants with baseline serum 25(OH)D concentrations ≥ 50 nmol/L and protein intakes ≥ 1.0 g/kg/day experienced significantly greater increases in appendicular muscle mass, the skeletal muscle index, and relative appendicular muscle mass following the intervention. These findings suggest that adequate vitamin D status and protein intake are important prerequisites for maximizing the anabolic response to nutritional supplementation in sarcopenic older adults, including older women [90]. Consistently, evidence from randomized controlled trials and meta-analyses indicates that vitamin D (800–2000 IU/day) combined with high-quality protein supplementation, particularly whey protein and leucine-enriched amino acid formulations, at doses of approximately 20–40 g per serving, is effective in improving muscle-related outcomes in older adults. This strategy holds particular relevance for postmenopausal women, who are at a higher risk of sarcopenia due to hormonal decline and reduced anabolic sensitivity [91,92,93,94]. A systematic review and meta-analysis of randomized controlled trials on sarcopenic adults, including older and postmenopausal women, reported that vitamin D plus protein intake significantly improved handgrip strength and sit-to-stand performance compared with to placebo, although effects on lean mass were more modest [32,95,96]. Protein supplementation consisted of whey protein, milk-based protein supplements, protein-enriched oral nutritional supplements, and essential amino acid mixtures (frequently including leucine-rich formulations) [32,95,96]. A clinical study explored formulas fortified with leucine and whey protein, combined with vitamin D3 supplementation, demonstrating improvements in appendicular muscle mass, lower-limb function, and overall physical performance in older women [97]. Another example comes from a network meta-analysis of nutritional interventions, involving older adults of both sexes, which highlighted that whey protein supplementation, alone or combined with vitamin D, is likely to enhance muscle strength and quality of life relative to usual care, providing moderate evidence for these interventions. Importantly, postmenopausal women often benefit the most due to age- and hormone-related muscle vulnerability [98,99]. A similar effect was observed when combining whey protein supplementation with vitamins D and E, leading to improved muscle strength and an increase in IGF-I and IL-2 in older sarcopenic adults, including older women [100]. Additionally, a recent meta-analysis reported that the combination of whey protein, leucine, and vitamin D can increase appendicular muscle mass in patients with sarcopenia [101]. These findings were partially derived from older and postmenopausal women, suggesting that this group may experience meaningful functional improvements due to increased sensitivity to protein and vitamin D interventions in the context of age-related anabolic resistance [101]. Furthermore, observational studies indicate that higher adherence to the Mediterranean diet is associated with greater muscle strength and a lower prevalence of probable sarcopenia in older adults [102]. The Mediterranean diet may influence musculoskeletal health through its anti-inflammatory and antioxidant components, combined with a higher intake of protein, vitamins (including vitamin D), and healthy fats, which collectively support muscle metabolism and physical function [102]. This protective effect is not attributable to a single nutrient, but rather to the synergistic interaction among high-quality proteins, anti-inflammatory lipids, antioxidants, and micronutrients. Intervention studies specifically testing the Mediterranean diet combined with vitamin D supplementation in sarcopenic populations are limited. However, some observational reviews, without distinguishing the specific effects of vitamin D, support the concept that vitamin D acts as part of a broader dietary synergy, contributing to muscle maintenance within an anti-inflammatory nutritional environment [103]. In the Mediterranean diet, this synergy is mainly supported by the combined presence of leucine-rich proteins, omega-3 polyunsaturated fatty acids (PUFAs), polyphenols, magnesium, and antioxidant vitamins [104]. Available studies do not provide detailed sex-stratified analyses, but older women, particularly postmenopausal women, represent a key high-risk subgroup. The findings are therefore highly relevant to women, who may benefit significantly from dietary strategies that simultaneously address protein insufficiency, vitamin D status, and chronic inflammation [103]. A critical aspect of the Mediterranean diet is its exceptionally high overall dietary quality, which correlates inversely with the incidence of frailty and muscle mass depletion in female aging populations. The high diet quality index of this program ensures an abundant, concurrent intake of magnesium, potassium, carotenoids, and vitamins C and E. This dense micronutrient environment works synergistically with the VDR axis to buffer skeletal muscle against oxidative stress and maintain myofibrillar structural integrity [102]. Furthermore, the Mediterranean diet offers sophisticated qualitative optimization of protein sources, prioritizing lean, high-biological-value proteins derived from poultry, fish, and dairy, balanced alongside plant-based proteins from legumes and nuts. This specific combination ensures a steady, rich supply of essential amino acids, particularly leucine, which acts as a direct chemical trigger for the mechanistic target of the mTORC1 pathway, the primary driver of muscle protein synthesis [103,104].

More recently, Venturini et al. [105] proposed that ketogenic diets and β-hydroxybutyrate (the primary ketone body) may exert potential anti-catabolic effects by reducing inflammation and suppressing proteolytic pathways. These mechanisms counteract muscle wasting, a condition highly prevalent in older women due to postmenopausal estrogen decline. However, the effectiveness of this dietary approach in managing sarcopenia depends strongly on maintaining adequate micronutrient status, particularly vitamin D. Concurrently, ketogenic approaches enriched with high-quality whey protein and branched-chain amino acids, especially leucine, may provide the necessary anabolic support to counteract age-related anabolic resistance. In fact, successful interventions in sarcopenic individuals may rely on the integration of vitamin D (1000–2000 IU/day) with leucine-rich amino acids and adequate micronutrient intake, rather than ketosis alone. Comparative evidence between dietary models remains insufficient, especially in older women and postmenopausal populations who are at a higher risk of vitamin D deficiency and accelerated muscle loss. Therefore, future well-designed intervention studies are required to establish optimal vitamin D supplementation strategies, define the most effective dietary patterns for sarcopenia prevention and management, and better understand possible sex-specific responses and long-term clinical outcomes [106].

## 6. Vitamin D and Physical Exercise Against Sarcopenia

Vitamin D status and physical activity are key factors associated with the prevention and management of sarcopenia, particularly in older and postmenopausal women, who are more susceptible to muscle loss.

- Mechanistic Rationale and Clinical Implications

Although resistance exercise is widely recognized as an effective strategy to counteract sarcopenia, its anabolic efficacy appears attenuated in older adults compared to younger individuals due to age-related anabolic resistance [107]. At the molecular level, vitamin D and resistance exercise synergistically boost muscle protein synthesis by inhibiting the TSC1/2 complex and activating Rheb-mediated mTORC1 signaling [108,109]. Conversely, metabolic stress from aerobic exercise activates AMPK, which dampens mTORC1 signaling.

**Figure 3:**
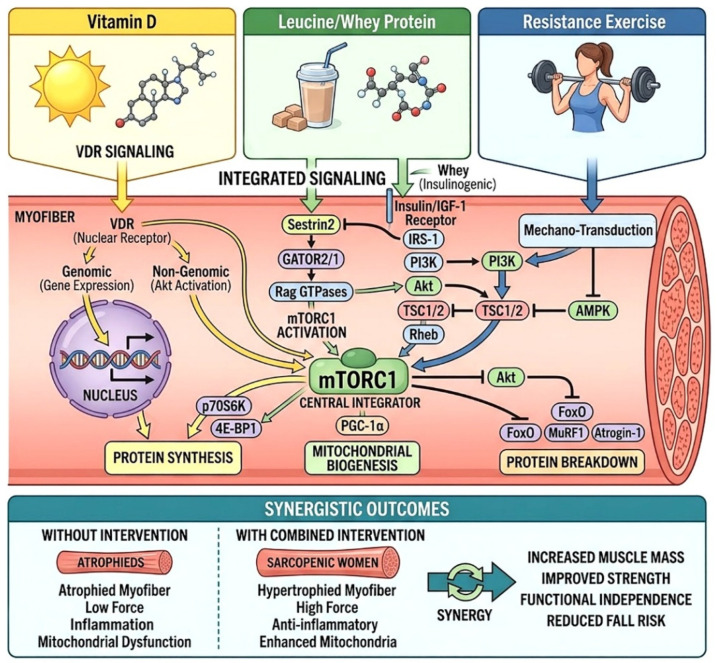
Schematic combined intervention of vitamin D, diet intake (whey protein/leucine), and resistance exercise: synergistic mechanism for counteracting sarcopenia in women. This figure was conceptualized using ChatGPT and manually reconstructed and finalized using Adobe Photoshop.

Beyond anabolism, the interplay between exercise and vitamin D can modulate inflammation and mitochondrial health. Their combination has been shown to suppress NF-κB signaling, reducing pro-inflammatory cytokines, while concurrently upregulating PGC-1α to drive mitochondrial biogenesis and reduce oxidative stress [76,109]. This mechanical synergy offers a plausible biological framework for optimizing functional outcomes in sarcopenic female populations, as schematized in Figure 3.

- Evidence from Trials in Older and Postmenopausal Women

Clinical evidence confirms that the functional benefits of these combined interventions are particularly relevant for postmenopausal women at high risk of sarcopenia and falls, though outcomes are often highly selective. In a randomized clinical trial by Uusi-Rasi et al. [110], structured exercise training combined with vitamin D supplementation significantly reduced fall risk and improved neuromuscular performance in older women, specifically those with low baseline vitamin D status. Similarly, Zhang et al. [111] reported that adding vitamin D to a resistance, balance, and walking program yielded superior improvements in SPPB scores and stair-climbing ability in older women compared to exercise alone. In overweight and obese postmenopausal women, Zaravar et al. [112] demonstrated that water-based aerobic training combined with vitamin D3 supplementation led to greater improvements in handgrip strength, gait speed, and balance than either intervention alone. Importantly, across these trials, improvements were predominantly functional and neuromuscular rather than structural, as changes in muscle mass and hypertrophy remained modest or inconsistent [111].

- Evidence from Mixed-Sex Older Adult Populations

Studies in broader, mixed-sex cohorts of older adults echo these findings, highlighting selective functional adaptations rather than uniform musculoskeletal reversals. A systematic review and meta-analysis by Antoniak and Greig [113] concluded that combining resistance training with vitamin D3 led to modest but significant improvements in lower-limb performance, mobility, and balance, while effects on muscle mass were inconsistent. Consistent with this task-specific pattern, Mesinovic et al. [109] found that a 24-week multimodal exercise and vitamin D3 regimen in vitamin D-deficient, overweight older adults improved selective measures like stair-climb time and waist circumference but failed to enhance global gait speed or overall physical performance compared to controls. Low-intensity, unsupervised, home-based exercise programs—such as those evaluated in the DO-HEALTH trial [114]—demonstrated minimal additive effects when combined with vitamin D, emphasizing that proper training intensity and supervision are critical. Furthermore, the clinical benefit appears strictly confined to populations with pre-existing deficiency. When multi-ingredient supplements (containing whey protein, leucine, and vitamin D) are integrated with physical exercise, some studies report significant increases in both muscle power and the appendicular muscle index [101,115]. However, other protocols, such as the 12-week program by Kazeminasab et al. [116], showed only modest improvements in knee extension and handgrip strength without significantly affecting overall muscular strength. These mixed data suggest that combined interventions generally serve to attenuate, rather than reverse, the progression of sarcopenia-related decline.

- Limitations of Current Evidence and Boundary Conditions

A major caveat in the current literature is that vitamin D supplementation does not consistently enhance exercise outcomes. The synergy is heavily dependent on program design and baseline nutritional status. For instance, a recent trial in elderly sarcopenic patients with baseline 25(OH)D < 50 nmol/L showed that vitamin D plus resistance exercise and thermotherapy led to significantly greater improvements in muscle function and inflammatory suppression compared to rehabilitation alone [117]. Conversely, a randomized clinical trial in older adults with normal baseline vitamin D levels found no significant additive effects on body composition, muscle strength, or inflammatory status [118]. The primary methodological gap across current literature remains the lack of consistent stratification according to baseline serum 25(OH)D status, which is essential to resolve existing discrepancies and target interventions effectively.

The effects of vitamin D supplementation and physical exercise in the older population, including women, are summarized in Table 2. The major methodological gap across these studies can be attributed to the lack of consistent stratification according to baseline serum 25(OH)D status.

## 7. Limitations and Critical Considerations

Despite the promising biological mechanisms linking vitamin D to muscle health, the clinical evidence gathered so far remains highly fragmented and controversial. Although many studies have reported beneficial effects of vitamin D supplementation on muscle strength, function, and sarcopenia-related outcomes, several critical factors and methodological heterogeneities must be considered to properly contextualize these conflicting findings:

- Differences in Baseline Vitamin D Status: A major limitation affecting the consistency of current trials is the lack of stratification based on initial serum 25(OH)D levels. Clinical responses appear to depend heavily on baseline status; beneficial effects are predominantly observed in individuals with severe, true baseline deficiency. Conversely, supplementing older adults who already possess sufficient or only mildly deficient levels yields negligible or null clinical improvements, as demonstrated by several studies.
- Variations in Supplementation Dose, Protocol, and Duration: Nutritional strategies across the analyzed literature vary dramatically, ranging from low daily maintenance doses to high-dose boluses (e.g., daily vs. bolus dosing), which can significantly influence the observed outcomes. Furthermore, trial durations span from a few weeks to up to 36 months. This marked discrepancy hinders the standardization of clinical protocols and limits the ability to assess long-term benefits, safety, and the exact therapeutic window required to elicit a meaningful musculoskeletal response.
- Heterogeneity in Sarcopenia Definitions and Outcome Measures: The lack of a unique consensus in defining sarcopenic status, combined with the diversity of adopted outcomes, such as muscle mass, strength, balance, or physical performance (SPPB scores, handgrip strength), heavily confounds data interpretation and complicates direct comparisons across studies. Current evidence suggests that vitamin D may selectively impact muscle quality, functional performance, and neurological gait parameters rather than inducing muscle hypertrophy or increasing overall muscle mass. Mixing these distinct physiological outcomes often dilutes the perceived efficacy of the intervention.
- Study Design and Synergistic Interventions: The therapeutic isolation of vitamin D represents another significant bias. Giving vitamin D alone to healthy, community-dwelling older adults often leads to no practical results, as shown by several large clinical trials. However, the clinical effectiveness of vitamin D increases significantly when it is part of a combined approach. Its impact becomes truly meaningful only when it works in synergy with resistance training (strength exercises) and specific nutrients, such as whey protein and leucine, which are essential to trigger muscle protein synthesis and promote muscle growth. Integrating these components is therefore crucial, as single-nutrient supplementation frequently dilutes the perceived efficacy of the intervention.
- Lack of Stratification and Sex-Specific Analyses: A significant limitation of the existing evidence is the scarcity of studies conducted exclusively on female populations, particularly postmenopausal women or adult women. Many included studies involve mixed populations of men and women or analyze older age groups without sex-specific stratification.
- Lack of Mechanistic Integration Between VDR and Estrogen Signaling in skeletal muscle: current studies do not provide clear evidence of a cross-talk or causal link whereby declining estrogen levels, such as those seen in postmenopausal women, lead to a significant reduction in VDR expression in skeletal muscle tissue. This represents a gap in the understanding of how hormonal fluctuations may influence vitamin D signaling pathways and muscle health, highlighting an area in need of further investigation.
- Potential Publication Bias: Finally, the current landscape of literature may be partially distorted by publication bias, where smaller trials reporting null or negative associations regarding vitamin D monotherapy fail to be published, potentially overemphasizing positive trends.

Overall, these interconnected factors contribute to the high heterogeneity and sometimes contrasting findings in the literature, underscoring the urgent need for more standardized, stratified, and rigorously designed clinical trials.

## 8. Future Directions

To overcome the limitations of current clinical and preclinical models, future research should leverage advanced translation platforms. The utilization of human primary cells and muscle biopsies remains essential to bridge the gap between bench and bedside. Furthermore, cutting-edge methodologies—such as three-dimensional (3D) skeletal muscle organoids and induced pluripotent stem cells (iPSCs)—offer unprecedented opportunities to model human muscle tissue and postmenopausal physiology in vitro without the confounding variables of animal models. Integrating these platforms with high-throughput “omics” technologies (including transcriptomics, proteomics, and metabolomics) will allow for a deeper characterization of the molecular signatures driving muscle wasting. Prioritizing mechanistic research through these innovative tools will be essential to map precise signaling alterations and identify novel therapeutic targets for sarcopenia.

## 9. Conclusions

This review highlights that vitamin D signaling may serve as a biological modulator of skeletal muscle health, mass, and functional capacity in women. However, the available clinical evidence remains highly heterogeneous, occasionally contradictory, and frequently lacks sex-specific granularity, particularly regarding muscle hypertrophy and long-term physical performance. Rather than representing a universally consistent therapy, vitamin D supplementation appears to be relevant primarily in women with a confirmed baseline deficiency or insufficiency. Accumulating data suggest that the therapeutic outcomes of vitamin D are not uniform; instead, they are highly likely to depend on a complex interplay of clinical variables, including the patient’s baseline nutritional status, the type and intensity of the exercise program, concurrent dietary protein intake, the total duration of the intervention, and the specific diagnostic criteria utilized to define sarcopenia. While combining vitamin D with structured physical exercise and targeted dietary protein intake is often proposed as a plausible strategy to mitigate age-related muscle decline, its definitive efficacy remains to be established. Ultimately, to resolve these ambiguities and formulate precise clinical guidelines, well-designed, long-term, sex-specific randomized controlled trials are required to carefully evaluate these compounding variables in aging female populations.

## Figures and Tables

**Table 1:** Serum levels of 25(OH)D.

	Serum 25(OH)D (nmol/L)	Serum 25(OH)D (ng/mL)
Optimal	>75	>30
Adequate	50–75	20–30
Insufficient	30–50	12–20
Deficient	<30	<12

**Table 2:** Summary of studies investigating the effects of combining vitamin D supplementation with physical exercise in postmenopausal and older women, including mixed-sex cohorts. Study design, population characteristics, vitamin D dosage, exercise interventions, and main outcomes are reported. (NR = not reported).

Study Design	Population	Exercise Program	Baseline Serum 25(OH)D Levels	Vitamin D Dose and Duration	Primary Outcome
Double-blind, placebo-controlled trial [109]	Overweight/obese older adults 50 participants (60% female) Age range: 50–80	Multimodal (resistance + aerobic + functional)	<50 nmol/L	4000 IU/day for 6 months	Physical function (gait speed, stair climb), body composition
Randomized clinical trial [110]	Older women 409 participants (100% female) Age range: 70–80	Supervised balance + strength training	NR	800 IU/day for 24 months	Falls incidence and physical performance
Scoping review of Randomized clinical trial [111]	Older adults 1483 participants (79.9% female) Age range ≥ 65	Resistance + balance + walking	<50 nmol/L in 2 studies 20–75 nmol/L in 6 studies >75 nmol/L in 1 study	800–2000 IU/day, for 2–24 months	SPPB score; stair-climbing performance
Systematic review and meta-analysis [113]	Older adults 792 participants (60–80% female) Age range ≥ 65	Primarily resistance training	NR	400–4000 IU/day for 3–24 months	Muscle strength, muscle mass, physical function
Randomized clinical trial [112]	Overweight/obese post-menopausal women 95 participants (100% female) Age range: 60–70	Water-based aerobic training	<50 nmol/L	1000 IU/day for 2 months	Muscle strength and physical function
Randomized clinical trial [114]	Older adults 1940 participants (63.8% female) Age range > 70 years	Home-based low-intensity strength program	NR	2000 IU/day for 36 months	No significant muscle mass increase
Multicenter Randomized clinical trial [115]	Older adults 110 participants (63.6% female) Age range > 65	Resistance + functional training	NR	200 IU/day for 4 months	Increase in appendicular muscle mass, strength and physical function
Systematic review and meta-analysis [116]	Adults 1675 participants (60–80% female) Age range: 21–83	Predominantly resistance training	<50 nmol/L in 28 studies	800–4000 IU/day from 2 to 12 months	Modest muscle strength increase
Randomized controlled clinical trial [117]	Older adults 140 participants (43.6% female) Age range > 65	Resistance training	<50 nmol/L	600 IU/day for 3 months	Improvement of muscle function
Randomized, double-blind study [118]	Older adults 26 participants (80.6% female) Age range > 60	Resistance training	>75 nmol/L	2000 IU/day for 4 months	No improvement in muscle function and strength

## Data Availability

No new data were created or analyzed in this study. Data sharing is not applicable to this article.
